# Pancreas sparing duodenectomy as an emergency procedure

**DOI:** 10.1186/1749-7922-4-19

**Published:** 2009-05-16

**Authors:** Piotr Paluszkiewicz, Wojciech Dudek, Kathryn Lowery, Colin A Hart

**Affiliations:** 1 Department of Surgery and Surgical Nursing, Medical University of Lublin, Lublin, Poland; 2 Department of Surgery, Pilgrim Hospital, Boston, Lincolnshire, UK

## Abstract

**Background:**

The operative techniques to close extensive wounds to the duodenum are well described. However, postoperative morbidity is common and includes suture line leak and the formation of fistulae. The aim of this case series is to present pancreas sparing duodenectomy as a safe and viable alternative procedure in the emergency milieu.

**Methods:**

Five patients underwent emergency pancreas sparing duodenal excisions. Re-implantation of the papilla of Vater or the papilla with a surrounding mucosal patch was performed in two patients. In one, the procedure was further supplemented with a duodenocholangiostomy, stapled pyloric exclusion and enterogastrostomy to defunction the pylorus. In another three patients, distal duodenal excisions were done.

**Results:**

In four patients, an uneventful recovery was made. One patient died following a myocardial infarction. The surgery lasted meanly 160 minutes with average blood loss of approximately 500 milliliters. The mean hospital stay was 12 days. Enteral nutrition was introduced within the 20 hours after the surgery. Long term follow-up of all surviving patients confirmed a good outcome and normal nutritional status.

**Conclusion:**

Based on the presented series of patients, we suggest that pancreas-sparing duodenectomy can be considered in selected patients with laceration of the duodenum deemed unsuitable for surgical reconstruction.

## Background

In recent years a pancreas-sparing duodenal excision (PSD) was introduced for the treatment of certain duodenal pathologies. This technique consists of total duodenal excision including the papilla of Vater with sparing of adjacent tissues, particularly pancreatic parenchyma and the distal biliary and pancreatic ducts.

PSD is less invasive than the formal pancreatico-duodenectomy and is indicated in selected cases of benign or traumatic lesions of the duodenum [1-3]. The benefits of this technique were described recently in patients with benign duodenal tumours [4,5]. Partial excisions of the duodenum to treat various malignant tumours involving the duodenal wall are also widely described in the literature [2,6-8]. The generous blood supply that remains, despite partially resecting the first two parts of duodenum, greatly assists in the success of closure by simple suturing. Under some circumstances it is necessary to resect the third and fourth part of the duodenum and reconstruct the duodeno-jejunal junction below the papilla [8].

The complex anatomy and common blood supply of the pancreatico-duodenal region both contribute to technically difficult and prolonged operations [9], therefore performing a PSD an emergency is considered only under specific conditions and is generally avoided. The emergency PSD (EPSD) is uncommonly described and rarely in patients suffering trauma [4,10]. The aim of this paper is to describe a series of five patients treated successfully in the emergency setting with pancreas-sparing duodenectomy as well as identify factors that may have contributed to the successful outcomes we have observed.

## Methods

### Patients

Five patients underwent emergency pancreas-sparing duodenectomies during 2002 – 2007. Data was retrospectively collected and analysed from inpatient records and outpatient documentation. The use of patients' records for the purpose of this article was approved by local Ethical Committee at Medical University of Lublin, Poland (decision number KE-0254/216/2008). The clinical features, duration of surgery, intra-operative blood loss, length of intensive care unit admission and total hospital stay were studied. The outcomes and complications were also reviewed.

### Surgical management

**Figure 1:**
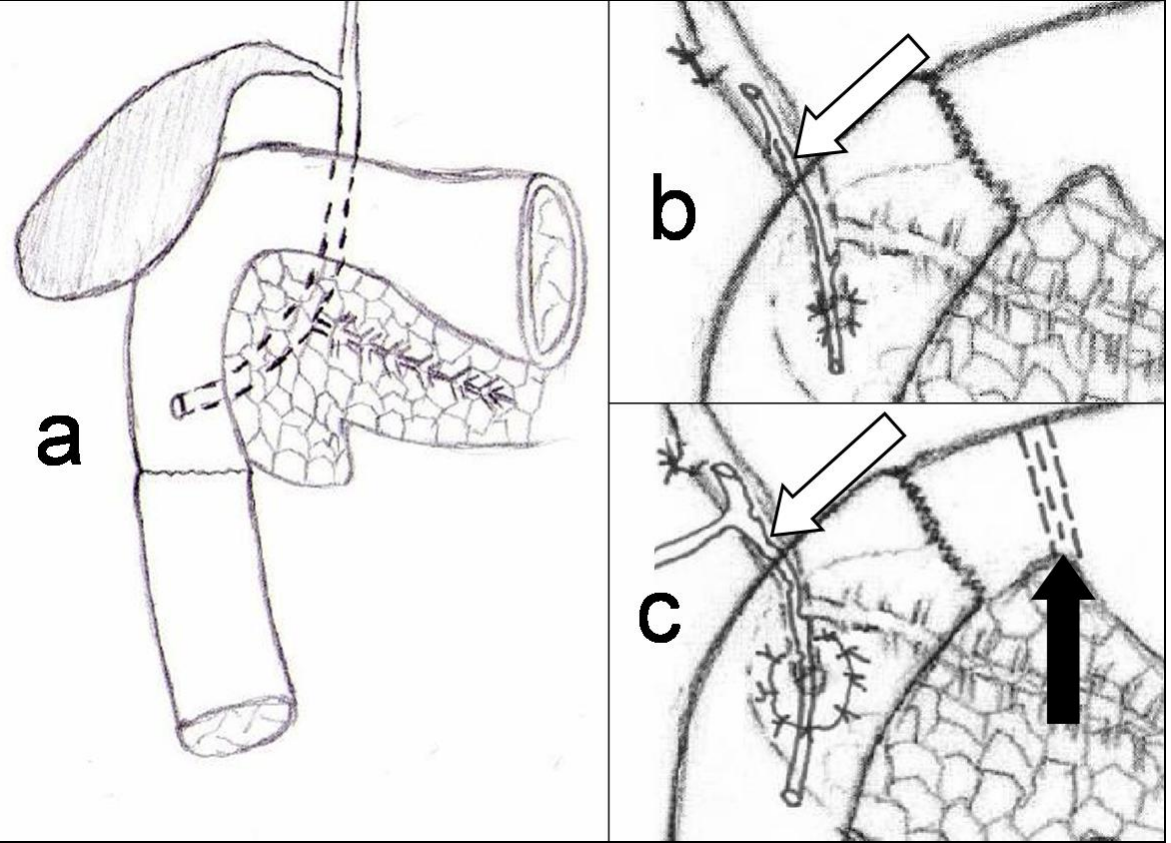
**Lacerations of D2-3 or D1-2-3 parts of duodenum not suitable for reconstruction with simple suture or Roux-en-Y closure. **Duodenal reconstruction was achieved by distal and total duodenectomy with sparing pancreatic parenchyma. The distal duodenectomy with the end-to-end junction between the duodenum and jejunum at approximately 1 cm below the papilla (a). Total duodenectomy with end-to-end anastomosis between the duodenal cuff and the jejunum (b, c). The papilla was implanted to the side of the jejunum with (c) or without mucosal islet (b). Biliary stent (marked by arrow) prevented postoperative stricture of the anastomosis due to oedema (b). Pyloric exclusion (black arrow) as well as the T-tube enterocholangiostomy (white arrow) were performed to prevent anastomotic leak. The adjunct enterogastrostomy was not present in the figure (c).

A xypho-umbilical laparotomy was performed in all cases. Extensive mobilisation of the duodenum using Kocher's manoeuvre was also supplemented by liberation of Treiz ligament. The upper jejunum was transected after division and ligation of duodeno-jejunal mesenteric flexure. The second (D2) and third (D3) part of the duodenum were divided carefully from the parenchyma of the head of the pancreas. Haemostasis was achieved via mono/bipolar diathermy and single haemostatic sutures of the pancreatic tissue. In three cases D2 was dissected 1 cm below the papilla of Vater (Figure 1a). In the remainder, both duodenal bulb and D2 were removed. In these latter two cases an anastamosis was formed between the isolated ampulla (Figure 1b) or surrounding mucosal patch to the side of a jejunal loop (Figure 1c). This was performed using absorbable polyfilament 4/0 interrupted sutures (Figure 1b,c).

An end-to-end anastamosis between the jejunum and duodenal cuff was performed using sero-muscular absorbable polyfilament 3/0 sutures. In one case the procedure was supplemented by a retrocolic gastroenterostomy, T-tube duodenocholangiostomy and stapled pyloric exclusion (Table 1, Figure 1c). The naso-jejunal feeding tube (8 Ch, 140 cm) as well as a naso-gastric decompression tube (12 Ch, 80 cm) was inserted intra-operatively in all cases.

**Table 1:** Clinical features and surgical strategy in the patients underwent pancreatic sparing duodenectomy as an emergency procedure

Patient N°	Sex	Age	Cause of surgery	Duodenal resection	Supplemented procedures
1.	M	57	Road traffic, blunt abdominal trauma, complex pancreatico-duodenal injury	partially D1, D2-4	enterogastrostomy, T-tube cholangioenterostomy, pyloric exclusion, cholecystectomy
2.	M	81	Gut bleeding, giant peptic ulcers of duodenum localised in D1 and D2/3 surrounded the papilla	partially D1, D2-4	bile stent inserted transpapillary
3.	F	72	Ischemic necrosis of jejuno-dodenal flexure	partially D2, D3-4	resection of the middle part (50 cm) of small intestine
4.	F	49	Foreign body (chicken bone) perforation of D3	partially D2, D3-4	none
5.	M	69	Gut bleeding, giant peptic ulcer localised in D2/3	partially D2, D3-4	none

### Post-operative management and outcome

Enteral feeding via the naso-jejunal tube was commenced on the first postoperative day directly after haemodynamic stabilisation. Gastro-intestinal protection (150 milligrams of ranitidine per day) was started 3 hours post-operatively and thromboembolic prophylaxis (0.6 millilitres of nadroparin per day – 11,400 anti Xa IU) was initiated 12 hours after surgery. The wide-spectrum antibiotics were administered for five post-operative days in all patients.

## Results

All cases were performed as emergency procedures. In two cases giant peptic ulcers were diagnosed at endoscopy. In both cases visualisation and control of the torrential duodenal bleeding was impossible (patients 2 and 5, Table 1). Two patients required the packed red cells transfusion due to extensive pre-operative bleeding (patients 2 and 5 on Table 2). Perforation of the duodenal wall was discovered (intra-peritoneal air collection on the CT-scans performed pre-operatively) in two further cases (patients 1 and 4, Table 1). In the final case multiple focal necrosis due to thromboembolic occlusion of the mesenteric arteries was revealed (patients 3, Table 1). Unfortunately, ischaemic necrosis of the duodeno-jejunal flexure with significant ischaemia of the third part of duodenum challenged the duodenal excision (Table 1).

**Table 2:** On-table data in patients underwent emergency pancreatic sparing duodenectomy

Patient N°	Pre-op pRBC transfusion^a^	Length of surgery (min.)	On-table blood loss (ml)	Peri-op pRBC transfusion^b^	Total intra-operative fluid transfusion (ml)
1.	none	160	400	none	2,000
2.	3 units	190	1,100	3 units	2,400
3.	none	100	300	none	1,000
4.	none	90	300	none	1,500
5.	2 units	140	400	none	1,500

Mean		136	500		1,700

The number of units of packed red blood cells (pRBC) transfused pre-operatively (^a^) or during first 24 hours after the commencement of the emergency pancreas sparing duodenectomy including on-table ingestion (^b^).

Three of five patients required concurrent procedures in addition to EPSD. One patient required a prophylactic T-tube cholangioenterostomy to prevent anastomotic leak (patient 1, Table 1, Figure 1c) supplemented by enterogastrostomy due to exclusion of pyloric transit. A second patient had a biliary stent inserted to prevent oedema and the subsequent development of an inflammatory stricture at the site of anastamosis between the ampulla and the jejunum directly after surgery (patient 2, Table 1, Figure 1b); a third required the resection of an ischaemic length of jejunum (patient 3, Table 1). Mean operative time was just over 2 hours and relatively insignificant on-table blood loss was achieved (Table 2). Intravenous transfusion of not more than 2.5 litres was required in any case.

Enteral feeding via a nasojejunal tube was introduced in all patients at first day post-operatively. Only in one case was such the nutritional support supplemented via the parenteral route (Table 3). The cumulative 7-days nitrogen balance was minimally negative. The positive nitrogen balance was observed only in one case nourished synchronously by enteral and parenteral routes. Oral feeding was commenced on the fourth post-operative day in the patient with pyloric exclusion. In the rest with a patent pylorus, a liquid diet was launched on the 6^th^–7^th ^postoperative day.

**Table 3:** Postoperative course and outcome of the patients who underwent emergency pancreatic sparing duodenectomy

	Patient N°				
	1.	2.	3.	4.	5.
Duration of tube feeding (days)	7	15	8	6	9
Parenteral nutritional support	none	none	12 kcal/kg/day (9 days)	none	none
The start of liquid diet *per os*	4	7	7	6	6
Cumulative nitrogen balance during first 7 days after surgery	-6 grams	-18 grams	4 grams	0 gram	-8 grams
ICU free days	9	23	12	9	9
Length of hospital stay	10	28	12	9	12
Complications	none	myocardial infarction	urinary infection	none	wound infection
Outcome	discharged	died in 28^th ^post day	discharged	discharged	discharged

The length of hospital stay varied from 9 to 12 days following surgery. In one patient, with previously known cardio-pulmonary history, sudden cardiac death on the 28^th ^post-operative day occurred. In this patient, however, no adverse gastrointestinal events were recorded post-operatively. Of the total hospital stay, over 75% was ICU-free. In one EPSD patient there was no requirement for an ICU admission.

## Discussion

We present this series of five patients with severe injury to the duodenum who underwent an emergency pancreas sparing duodenectomy in complex clinical circumstances where normally such extensive surgical procedures would usually be contraindicated. Two patients required a resection of the all (D1-4) parts of duodenum and other three of the distal duodenum (D2-4). The decision-making process was guided in all cases by the wound healing of the reconstructed duodenal wall. Various reconstruction techniques including simple suture, Roux-en-Y closure or duodenal resection [11,12] were all considered. Unfortunately, the lacerated third part of duodenum in all five cases limited duodenal sparing surgery due to its insufficient blood supply. This has been confirmed using light spectroscopy [13]. Any anastamosis performed in such insufficiently perfused tissues are of course associated with a high incidence of postoperative complications including enteric leak, strictures and secondary sepsis. Thus, in the case of such extended duodenotomies associated with difficulties in duodenal wound closure or insufficient blood supply, duodenal excision may provide a viable alternative.

The successful outcome of EPSD with mortality rate of less than 1% (2/53) was recently presented in the group of traumatic patients who underwent EPSD or duodenal resection with primary anastamosis due to complex, blunt or penetrating, duodenal trauma (Table 4) [14-23]. In one of our patients the traumatic injury of the duodenum was associated with only superficial tears of pancreatic tissue without any marked additional injuries. In this patient, the reconstruction performed after the emergency duodenectomy was supplemented by the T-tube enterocholangiostomy, pyloric exclusion and enterogastrostomy. During the early post-traumatic period bypassing pyloric transit protects the complex suture lines in the duodenal wall [24,25]. In our opinion, the use of a 3-row linear stapler for pyloric exclusion is the simplest, fastest and most effective technique in pancreatico-duodenal surgery. In addition to the stapled pyloric exclusion, the T-tube duodeno-cholangiostomy controls duodenal output, removes corrosive duodenal content and decreases the intra-duodenal pressure [26]. The supplementation of pyloric exclusion by a truncal vagotomy in experimental studies has been shown to protect the mucosal layer from massive inflammation [27]. Recent experience demonstrates that truncal vagotomy may be replaced by intravenous administration of histamine receptor antagonists. Intravenous histamine receptor antagonists have been introduced in many centres in those patients suffering severe trauma or extended surgery as a preventative measure against gastro-intestinal bleeding and marginal ulcer formation [28]. These findings suggest that EPSD may be considered in some patients with isolated duodenal trauma.

**Table 4:** The pancreatic-sparing duodenectomy (PSD) and duodenal resection with primary anastamosis (DR) after blunt and penetrating injuries reported in the literature

			Type of injury			
					
Author	Operative management	N° of cases	blunt	penetrating	Morbidity	Mortality
Chung [14]	PSD	1	1	0	wound infection	0
Maher [4]	PSD	5	0	5	1/5 post-op bleeding	0
Yadav [10]	PSD	3	3	0	2/3 wound infection, burst abdomen, acute renal failure	0
Nagai [9]	PSD	1	not reported		not reported	0
**Total**	**PSD**	**10**			**4/10**	**0/10**

Huerta [15]	DR	5	1	4	not reported	0
Velmahos [16]	DR	11	not reported		4/11 included duodenal leak, abdominal abscess, wound infection, GI-bleeding, pancreatic fistula, pancreatitis, respiratory failure	0
Talving [17]	DR	7	0	7	1/7 duodenal leak	1/7
Ruso [18]	DR	3	0	3	not reported	0
Alessandroni [19]	DR	2	2	0	1/2 duodenal leak	1/2
Jurczak [20]	DR	4	not reported		not reported	0
Singh [21]	DR	1	1	0	not reported	0
Kline [22]	DR	4	0	4	not reported	0
Cogbill [23]	DR	6	not reported		1/6 intra-abdominal abscess	0
**Total**	**DR**	**43**			**7/43**	**2/43**

In one of presented patients the biliary stent was inserted to prevent the oedema and secondary stricture of the entero-biliary junction. In this particular case over 2/3 of the circumference of a papilla was surrounded by the peptic ulcer. Therefore we inserted the stent after excising the narrowed papilla below the pancreatico-biliary confluence in the ampulla. The proper outflow of the biliary and pancreatic contents following a surgery of the papilla is crucial in prevention of postoperative septic cholangitis and may be achieved by insertion of a biliary stent [29]. The outflow of the pancreatic juice via the wide pancreatico-ampullar junction was observed on table during catheterisation of Virsung duct with the 6F silastic catheter. In patients with narrowed pancreatico-ampullar junction, the additional pancreatic stent is recommended for prevention of acute and recurrent pancreatitis [30].

Uncontrolled gastrointestinal bleeding in two cases was treated successfully by EPSD after endoscopic intervention failed. The extended duodenotomy performed during inspection for bleeding sites created the necessity of complex reconstruction of D2-3 parts of the duodenum. In these two cases, D2-4 parts of duodenum were excised due to the compromised blood supply to the duodenal suture lines. The surgical cessation of bleeding is currently very rarely in use; only in patients with persistent or recurrent bleeding resistant to endoscopic or endovascular haemostatic techniques [31]. Thus in some special conditions an extended enterotomy to the duodenal lumen for localisation of the atypical bleeding sites is indicated. After haemostatic control is reassumed, the closure of the duodenum is sometimes precarious, especially when the suture line is localised near D2/3 or directly on its horizontal part (D3). Additionally, the intra-luminal pressure in infrapapillary region of duodenum reaches approximately 10 kPa and may be an important factor conditioning healing process [32]. Thus the intestinal loop decompression lowers the intra-luminal pressure and prevents the leak from suture-line [33].

The described surgical procedures resulted in good outcomes in four patients and although one patient suffered a terminal myocardial infarction at day 28, no adverse gastrointestinal events were recorded postoperatively. EPSD appears complex however the fact that it may be successfully applied in the emergency setting as a one-step and definitive surgical procedure makes it a very promising alternative to other less comprehensive procedures.

In all cases presented in this paper, the blood loss associated with EPSD itself was generally limited. Only in one patient with gastrointestinal haemorrhage were packed red cells required. This particular patient had a history of coronary disease and required a maintained haemoglobin level of above 10 g/dL for reducing strain on the heart through lowering tachycardia, improving anaemia and correcting of base-acid balance. Our group believe that careful surgical technique and the avoidance of any required blood resuscitation reduced both the risk of postoperative morbidity and improved outcome. The benefits of restricting blood transfusions have been described more recently in various clinical conditions [34].

Nasojejunal feeding tubes were introduced in all patients for early postoperative enteral nutrition. This nutritional support reduces septic events by maintaining integrity, limiting transmigration of bacteria, accelerates return of the bowel peristalsis and influences on inflammatory response during earliest days after surgery. Additionally, nutritional support shortens the length of stay both in the hospital and in ITU [35]. Only in one patient was enteral nutrition supplemented parenterally due to insufficient diet tolerance. The diet tolerance and possibility of enteral feeding lower the risk of hyperglycaemia, overfeeding and cause fewer complication than parenteral route [36].

## Conclusion

In conclusion we suggest that emergency pancreas sparing duodenectomy is a viable option in those patients with complex duodenal pathology when the effectiveness of classical surgical techniques is uncertain. Despite the successful outcome in this short series of patients who underwent emergency duodenectomy, further studies are indicated to fully evaluate this technique.

## Abbreviations

PSD: (Pancreas-Sparing Duodenectomy); EPSD: (Emergency Pancreas-Sparing Duodenectomy); ICU: (Intensive Care Unit).

## Competing interests

There are no competing interests.

The authors have no actual or potential political or financial interest in the publication of this paper in terms of material, information or techniques described. The authors have received no financial incentive to contribute to this paper.

The authors certify no commercial associations that may pose a conflict of interest in connection with the submitted article.

## Authors' contributions

PP – Study conception and design, analysis and interpretation of data, drafting of manuscript, critical revision.

WD – Acquisition of data, analysis and interpretation of data, drafting of manuscript.

KL – Analysis and interpretation of data, critical revision.

CAH – Analysis and interpretation of data, drafting of manuscript, critical revision.

All authors read and approved the final manuscript.
